# Splenic Subcapsular Hematoma Complicating a Case of Pancreatitis

**DOI:** 10.7759/cureus.9034

**Published:** 2020-07-06

**Authors:** Aveek Mukherjee, Raisa Ghosh, Sugirdhana Velpari

**Affiliations:** 1 Internal Medicine, Rutgers Robert Wood Johnson Medical School/Saint Peter's University Hospital, New Brunswick, USA; 2 Gastroenterology and Hepatology, Rutgers Robert Wood Johnson Medical School/Saint Peter's University Hospital, New Brunswick, USA

**Keywords:** spleen, pancreatitis, subcapsular, splenic hemorrhage, splenic hematoma, splenectomy, laparotomy, alcohol use

## Abstract

Splenic subcapsular hematoma is a rare complication of pancreatitis. The splenic vessels and the pancreatic tail lie close together in the lienorenal ligament. The pathologies in the pancreatic tail may occasionally affect the spleen resulting in splenic vein thrombosis, arterial pseudoaneurysm, subcapsular splenic hematoma, and splenic rupture. A 40-year-old male with a history of alcohol abuse and alcohol-induced pancreatitis presented with severe epigastric abdominal pain and was diagnosed with pancreatitis. Later during hospitalization he became dyspneic and hemodynamically unstable, with acute anemia requiring blood transfusion. An abdominal CT with angiography (CTA) revealed a splenic subcapsular hematoma with active bleeding which was managed by urgent exploratory laparotomy and splenectomy. Due to its rarity, diagnosis of splenic hematoma in pancreatitis is challenging with rapid identification and intervention being key to management.

## Introduction

Pancreatitis and its complications remain a leading cause of healthcare utilization in the United States [1]. Splenic complications in pancreatitis are uncommon, with subcapsular hematoma being extremely rare with an incidence of only 0.4% [2]. Here we present the case of a 40-year-old male patient presenting with acute pancreatitis associated with a large subcapsular splenic hematoma, who was hemodynamically unstable and underwent surgical management. The patient recovered uneventfully.

## Case presentation

A 40-year-old male patient with a past medical history of alcohol abuse and alcohol-induced pancreatitis presented to the ED with severe epigastric abdominal pain for two days. The pain was described as severe, throbbing in nature, continuous, and radiating from the epigastrium to the left hypochondrium. It was associated with persistent nausea, without any vomiting and loss of appetite. There was no history of hematemesis or melena. No fever or chills were reported. The patient had been discharged a week prior, after being hospitalized for 10 days due to an attack of acute pancreatitis brought on by excessive alcohol consumption. Following discharge, the patient had resumed his consumption of alcohol daily. No abdominal trauma was reported.

**Figure 1:**
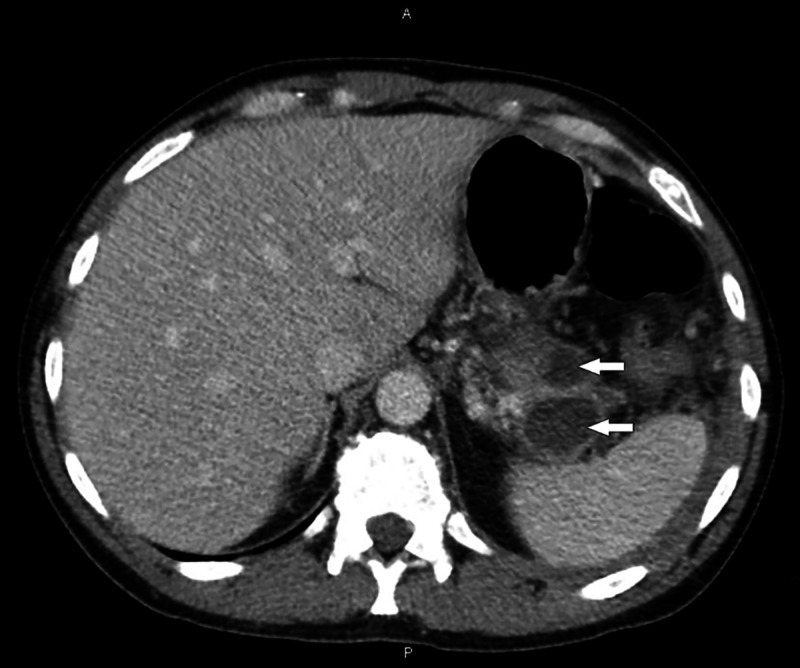
Previous CT scan of abdomen showing two pseudocysts in the pancreatic tail (white arrows).

On evaluation, his vital signs were noted to be stable. He was afebrile, oxygen saturations at 98% on room air, respiratory rate was 18 breaths per minute, pulse was 88 beats per minute, and blood pressure was 138/75 mmHg. The patient appeared very uncomfortable and was stooping forward while sitting. Abdominal examination revealed no distension, but marked tenderness with guarding was elicited at the epigastrium and left hypochondrium, without any rebound tenderness and bowel sounds were hypoactive. Laboratory investigations showed elevated white blood cells (12.1 x 109/L, normal 4-11 x 109/L), lipase (447 IU/L, normal 10-140 IU/L), amylase (360 IU/L, normal 20-110 IU/L), and alkaline phosphatase (147 IU/L, normal <110 IU/L). Also, hemoglobin was 138 g/L with a hematocrit of 40.2%, with normal total bilirubin, aspartate aminotransferase (AST), and alanine aminotransferase (ALT) at 0.4 mg/dL, 18 IU/L, and 22 IU/L respectively. Chest X-ray revealed normal cardio-pulmonary study. Only a week prior, his abdominal CT had revealed two pseudocysts in the pancreatic tail, along with evidence of acute pancreatitis, hence it was not repeated (Figure 1).

The patient was admitted to the medical floor and management was initiated for acute pancreatitis with nil per oral orders, IV rehydration, and IV opiate analgesics. By the second day, the patient noted mild improvement in his symptoms and clear liquids were started, which he tolerated well. We intended to advance diet as tolerated by the patient. Alcohol use cessation was discussed at length and support resources provided.

**Figure 2:**
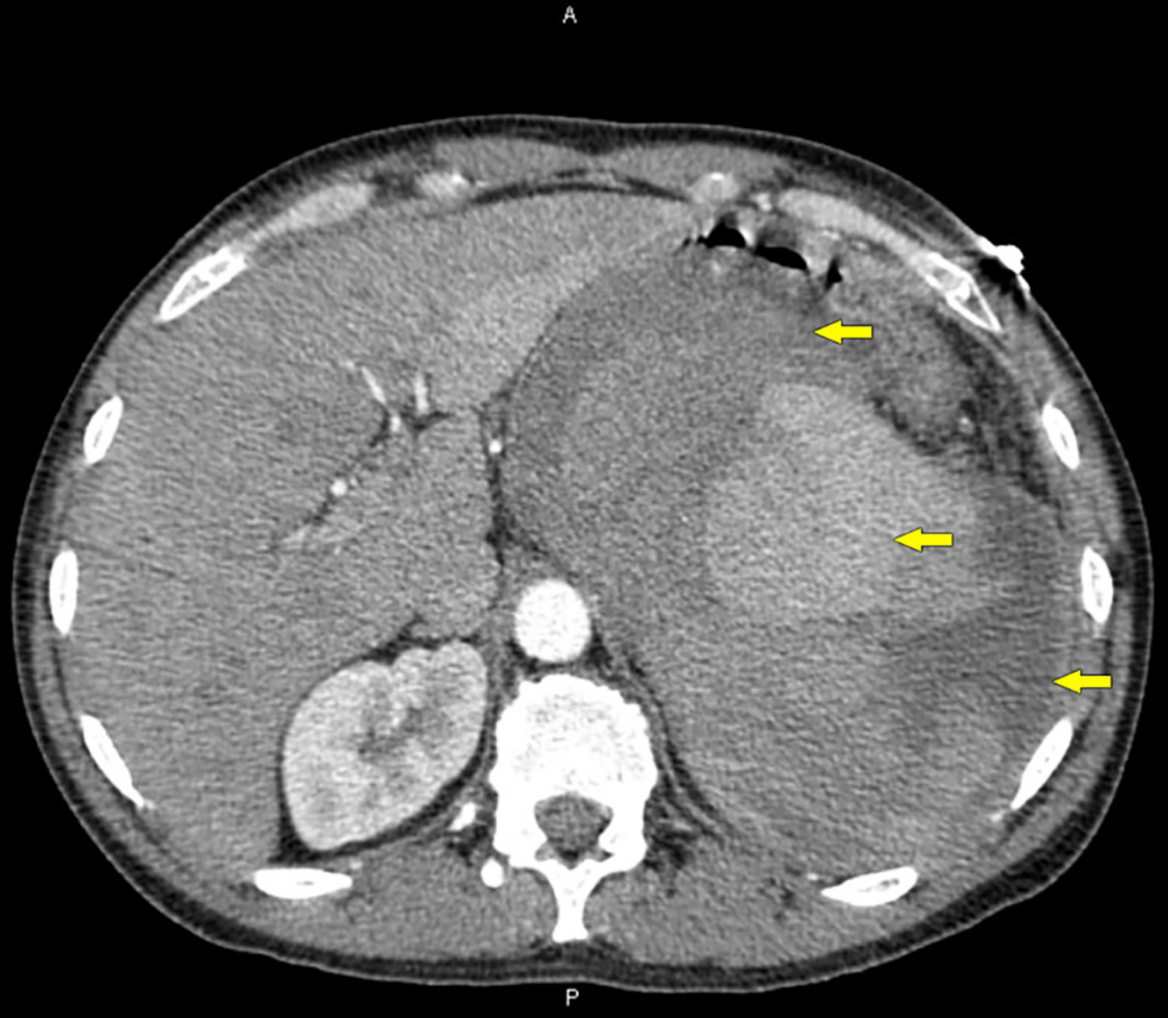
New CTA of abdomen showing huge variegated hematoma (yellow arrows) suggesting recent hemorrhage. CTA, CT with angiography

However, early on the third day, the patient complained of worsened abdominal pain, now localized more to the left hypochondrium and also had referred pain at the left shoulder. He became more nauseous and could no longer tolerate even liquids. He continued to be uncomfortable and in severe pain despite judicious IV analgesia. A few hours later, the patient suddenly became dyspneic and hypotensive and had to be shifted to the ICU for stabilization. Repeat investigations revealed a precipitous drop in his hemoglobin from 136 to 71 g/L (normal 135-155 g/L), but no overt signs of bleeding were found. This raised the concern for a concealed hemorrhage, possibly an intra-abdominal hemorrhage. Packed red blood cells were transfused and an urgent surgical consultation was called. A stat repeat abdominal CT with angiography (CTA) now revealed a large splenic subcapsular hematoma measuring 17.3 cm x 12.0 cm x 12.5 cm, communicating with a pseudocyst in the pancreatic tail, with areas of attenuation suggesting recent hemorrhage (Figure 2). Furthermore, it also revealed a blush of contrast extravasation in the distal splenic artery suggesting active bleeding.

The findings were discussed with the patient and he consented to surgical management. He underwent urgent exploratory laparotomy with splenectomy. Intraoperative findings confirmed erosion of a pancreatic pseudocyst into the splenic capsule as well as active bleeding. Hemostasis was successfully secured and splenectomy performed. After an uneventful procedure, the patient was transferred to the postanesthesia care unit where he experienced an uncomplicated recovery. He was discharged a week later. On follow up a month later, the patient was doing well with resolved abdominal pain and hemoglobin stable at 140 g/L.

## Discussion

The incidence of pancreatitis is increasing and remains a leading cause of hospital admissions with a gastrointestinal cause [1, 3-4]. Gallstones followed by alcohol remain the most common causes of pancreatitis, accounting for almost 70% of the cases [3].

The splenic vessels and the pancreatic tail lie close together in the lienorenal ligament, while entering the splenic hilum [4-6]. Therefore, pathologies in the pancreatic tail may occasionally affect the spleen, as depicted by the association of pancreatic tail pseudocysts and necrosis with splenic complications [2, 6]. Splenic complications of pancreatitis include splenic vein thrombosis, arterial pseudoaneurysm, subcapsular splenic hematoma, and splenic rupture [2, 4, 6]. Pathophysiology may include injury from extravasated pancreatic enzymes damaging the splenic capsule or parenchyma or blood vessels, causing splenic vein thrombosis, arterial pseudoaneurysm or hemorrhage, even splenic infarction [4-6]. An enlarging pseudocyst may also injure the splenic parenchyma or disrupt the splenic hilum directly, giving rise to complications [5-6]. Though the incidence of spontaneous splenic hemorrhage is rare, it is associated with significant morbidity and mortality [2, 6].

Patients with a splenic hematoma commonly present with upper abdominal pain, sometimes radiating to the left shoulder or back (Kehr's sign), often with a drop in hemoglobin [4, 6]. Due to the nonspecific presentation, abdominal CT and MRI studies are crucial for diagnosis, although MRI is preferred due to the better delineation of soft tissue structures [4, 6]. In our patient, the splenic hemorrhage was heralded by the patient’s hypotension, along with the left hypochondriac abdominal pain referred to his left shoulder. The diagnosis was later confirmed by CTA.

Management of this rare condition is controversial. Hemodynamically stable patients may often be managed conservatively, or with procedures such as splenic artery embolization or ultrasound-guided percutaneous drainage [4-5, 7-9]. However, in unstable patients, surgery remains key and can be lifesaving [4, 6, 10].

## Conclusions

Due to its rarity, the diagnosis of splenic hematoma in pancreatitis is challenging and requires a high index of suspicion. Rapid identification and intervention are critical to ensure an optimal outcome, with hemodynamic stability often deciding the mode of management. As the incidence of pancreatitis continues to rise, physicians should be aware of the complications for timely and effective management.
